# Recognition and Classification of Ship Images Based on SMS-PCNN Model

**DOI:** 10.3389/fnbot.2022.889308

**Published:** 2022-06-13

**Authors:** Fengxiang Wang, Huang Liang, Yalun Zhang, Qingxia Xu, Ruirui Zong

**Affiliations:** ^1^College of Electronic Engineering, Naval University of Engineering, Wuhan, China; ^2^Institute of Noise & Vibration, Naval University of Engineering, Wuhan, China; ^3^College of International Studies, National University of Defense Technology, Changsha, China

**Keywords:** image classification, multi-scale, CNN, ship images, ResNet

## Abstract

In the field of ship image recognition and classification, traditional algorithms lack attention to the differences between the grain of ship images. The differences in the hull structure of different categories of ships are reflected in the coarse-grain, whereas the differences in the ship equipment and superstructures of different ships of the same category are reflected in the fine-grain. To extract the ship features of different scales, the multi-scale paralleling CNN oriented on ships images (SMS-PCNN) model is proposed in this paper. This model has three characteristics. (1) Extracting image features of different sizes by parallelizing convolutional branches with different receptive fields. (2) The number of channels of the model is adjusted two times to extract features and eliminate redundant information. (3) The residual connection network is used to extend the network depth and mitigate the gradient disappearance. In this paper, we collected open-source images on the Internet to form an experimental dataset and conduct performance tests. The results show that the SMS-PCNN model proposed in this paper achieves 84.79% accuracy on the dataset, which is better than the existing four state-of-the-art approaches. By the ablation experiments, the effectiveness of the optimization tricks used in the model is verified.

## Introduction

In the military field, ship image classification is used to conduct precise strikes against hostile targets and important to carry out counter-terrorism missions. In the civilian field, ship image classification can assist relevant departments in maritime traffic control, search and rescue, and anti-smuggling activities. Therefore, ship image classification has broad applications and technical requirements in both military and civilian fields.

Currently, there are four types of maritime target images: radar images, remote sensing images, infrared images, and visible light images. Radar (Jiang et al., 2021; Tang et al., 2021) image recognition is all-weather and daylong, which means that it is not easily affected by light and weather. Its mainstream approach is extracting and classifying the features of radar echo signals, so as to achieve autonomous deep feature extraction of the data. Remote sensing (Yang et al., 2014, 2017) image recognition is extracting geometric features such as length and contour of targets in high-resolution SAR remote sensing images, so as to enhance SAR image recognition capability. Infrared image recognition can work in a long distance, which can penetrate thick fog and work day and night.

For visible images, traditional image recognition and classification use techniques including pixel-level edge detection, genetic algorithm, and support vector machine (SVM) classification. Atsuto and Kazuhiro (2004) proposed a multi-frame image processing algorithm to extract contours as basic features of targets for vector analysis, achieving good recognition performance. Xu et al. (2017) designed a multi-level discrimination method based on the improved entropy and pixel distribution with multi-scale and multidirectional decomposition of high-frequency coefficients, which can effectively resist background interferences and improve recognition accuracy and efficiency. Yang and Kim (2012) integrated SAR and automatic identification system (AIS) datasets as one system that can display the position, size, and classification of ships on SAR images. Enriquez de Luna et al. (2005) proposed a silhouette-based decision support system for ship image classification, using an evolved version of the Curvature Scale space (CSS) to improve recognition accuracy.

In the recent years, convolutional neural networks (CNNs) have gradually been widely applied in visible light image classification and recognition. A series of classic CNN models, including AlexNet, VGG, GoogLeNet, ResNet, DenseNet, and so on, stand out in the ImageNet Large Scale Visual Recognition Challenge (ILSVRC), which covers various fields such as image classification and target detection.

The AlexNet (Alex et al., 2017) model is the first multi-layer CNN with five convolutional layers, three fully connected layers, and a max-pooling layer. This state-of-the-art model is a landmark of CNN model. The proposal of the VGG (Simonyan and Zisserman, 2014) model made the 3 × 3 convolution filters mainstream and improved the accuracy based on the AlexNet, achieving the state-of-the-art results and accelerating further research on the use of deep visual representations in computer vision. Since 2014, the GoogLeNet series has begun to emerge. GoogLeNet (Christian et al., 2014) proposed the inception convolutional neural network that started application of 1 × 1 convolution, reducing the amounts of computation and improving the utilization of computational resources. Its improved model GoogLeNet-V2 (Sergey and Christian, 2015) replaces the 5 × 5 convolution with two layers of 3 × 3 convolution, and the batch normalization proposed in this paper is widely used in deep neural networks. The Inception-V3 model proposed by GoogLeNet-V3 (Christian et al., 2015) achieved the state-of-the-art in the 2015 ILSVRC classification challenge. This model summarized four guidelines for network model design and three optimization tricks to effectively reduce the number of parameters and improve computational efficiency. GoogleNet-V4 (Szegedy et al., 2017) proposed the Inception-V4 model to summarize and integrate. It designed the Inception-ResNet network, which introduces residual connections into the GoogleNet series. The ResNet (He et al., 2016) model is designed with residual connection module, which is easier to optimize and has lower computational complexity and higher accuracy. The ResNeXt (Xie et al., 2017) model improved based on the ResNet model which integrated the ideas of VGG, ResNet, and Inception series. ResNeXt proposed cardinality index to measure complexity, winning the runner-up in the 2016 ILSVRC classification challenge. The DenseNet (Huang et al., 2017) model can effectively alleviate gradient disappearance and strengthen feature propagation, and therefore, it can outperform ResNet in all aspects on Cifar-10, SVHN, and Cifar-100 standard datasets. Besides, it can reduce the number of parameters by half with the same accuracy. The Senet (Jie et al., 2017) model focuses on the channel relationship and introduces attention mechanism into the convolutional neural network, which can adaptively recalibrate channel-wise feature to improve performance.

Convolutional neural networks are also widely applied in speech recognition (Partha et al., 2020; Pradeep and Nirmaladevi, 2021; Yang et al., 2021), medical diagnosis (Seo and Kim, 2020; Toktam et al., 2020; Mustaqeem, 2021), biometrics (Alay, 2020; Sadasivan et al., 2020; Mekruksavanich and Jitpattanakul, 2021; Mohaghegh and Payne, 2021), and other fields. The exploration of convolutional neural networks in other fields provides a reference for us to use CNN for ship image recognition (Cazzato et al., 2020). Jeon and Yang (2021) proposed a classification model integrating CNN and KNN, which classifies from dual-polarization data with 10-m pixel distance to improve the efficiency of ship classification. Li et al. (2021) contribute to intelligent ship vision system by traditionally integrating image processing with machine learning and using target detection method based on CNN. Julianto et al. (2020) proposed a modified method using CNN to classify a patrol vessel dataset and achieved great results. Chen et al. (2020) combined the improved generative adversarial network (GAN) and convolutional neural network, proposing a small ship detection method, which significantly improved the accuracy and robustness of the results. Zhao et al. (2020) used the improved AlexNet convolutional neural network for deep feature extraction of ship images, which significantly improved the classification performance. Xu et al. (2020) proposed a detection method combining visual saliency and a convolutional neural network cascade, which can effectively improve ship detection accuracy and efficiency. Based on the pixel-level fusion of visible and infrared bispectral images, Gao (2020) integrated the algorithms that include image preprocessing, image smoothing, and anti-cloud interference to achieve the detection of ship targets in complex land and sea backgrounds. Ren et al. (2019) proposed a CNN framework with fewer layers and parameter. This method used the Softmax function to classify different ship types and achieved good results. Li et al. (2019) proposed a method to learn discriminative features through supervised learning and good classification performance and built two small optical ship image datasets to verify that results of this method. Hou et al. (2019) proposed a ship detection method based on the visual attention enhanced network to process optical remote sensing images, which improved the recognition accuracy and the detection performance. Endang and Agfianto (2019) combined the CNN-ZFNet architecture and the random forest method to extract the so-called best features defined in the paper and improved the ship image classification accuracy. Shao et al. (2019) proposed a saliency-aware CNN framework for ship detection, comprising comprehensive ship discriminative features. Makantasis et al. (2016) proposed that a methodology fuses a visual attention method that exploits low-level image features appropriately selected for maritime environment. Dong et al. (2018) proposed a hierarchical detection method that finishes the automatic ship detection well. Sobral et al. (2015) proposed a double-constrained robust principal component analysis (RPCA) to improve the object foreground detection in maritime scenes.

By now, various methods to solve the classification and recognition problem of ship targets by convolutional neural networks do not focus on the differences between the grain of ship images. We found that the differences between categories of ships are the hull structure, and the differences between ships of the same category are the equipment and superstructures. Therefore, for the differences in the hull structure between different categories of ships, we should use convolution kernels with larger receptive fields, which is conducive to extracting features such as hull structures that occupy large pixel areas. At the same time, for small differences between diverse ships of the same category, we should use convolution kernels with smaller receptive fields to extract fine features such as ship equipment and superstructures.

Based on the above ideas, we proposed the SMS-PCNN model to classify ship images. The model extracts the feature of images in different sizes by parallelizing convolution branches with different receptive fields. To enhance the speed of converge and avoid overfitting, three optimization tricks are introduced including enhancing specific ship dataset applications, improving training learning rate, and avoiding overfitting. Then, we collected 5,062 ship images of 12 categories on the Internet for the experiments to test the algorithm performance of the SMS-PCNN model and the effectiveness of three optimization tricks.

An outline of the paper is as follows. Section Introduction briefly introduces the research background, technical status quo, faced challenges, and the innovations of this paper. Section SMS-PCNN Model gives a detailed introduction to SMS-PCNN model, which uses three parallel CNN branches to extract the features from different receptive fields, and then classifies the feature maps based on the multilayer perceptron neural networks. Moreover, three optimization tricks of SMS-PCNN model to optimize network performance are also introduced in this chapter. Section Experiment tests the performance of the proposed SMS-PCNN model using the ship image dataset. Besides, a series of comparison experiments were also conducted to test the performance of algorithms using other state-of-the-art approaches as references. Besides, a series of ablation experiments are also conducted to verify the effectiveness of three optimization tricks used in the model. In addition, we conduct experiments on Branch1-CNN model only using 3 × 3 convolution to demonstrate the necessity of designing a multi-branch parallel structure. By comparing the accuracy of both experiments, we demonstrate that designing of a multi-branch parallel structure has better feature extraction capabilities for ship images than other. Section Conclusion summarizes the content of the paper.

## SMS-PCNN Model

This chapter presents the SMS-PCNN model proposed for the ship dataset and optimization tricks used by the model. SMS-PCNN uses three parallel CNN branches to extract feature maps from different receptive fields and then classifies the feature maps based on a multilayer perceptron neural network. This chapter is divided into two sections. The first section introduces the network architecture of the SMS-PCNN model in two levels. It introduces the basic single-branch architecture design for implementing the feature map extraction function in the SMS-PCNN model and then presents the SMS-PCNN model architecture design for integrating three single-branch CNNs with different receptive fields. The second section focuses on three optimization tricks for optimizing the network performance, including enhancing specific ship dataset applications, improving training learning rate, and avoiding overfitting.

### Model Architecture

This section is divided into two parts. The first part introduces the basic single-branch architecture design of SMS-PCNN. In this part, basic single-branch in parallel CNNs is used as an example, and the data processing procedure of RGB images after being inputting to each branch is introduced in a systematic way. In the second part, the overall network architecture of SMS-PCNN is presented. This part details the process of fusing feature maps from different single-branch CNNs and classifying the input images based on multilayer perceptron neural networks.

#### Basic Single-Branch Architecture of SMS-PCNN Model

**Figure 1:**
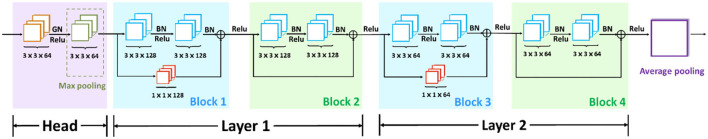
Network architecture of *Branch1*.

The SMS-PCNN model consists of three parallel CNN branches with different receptive fields. The three CNN branches have similar structures, so the first branch in the single-branch benchmark architecture network is taken as an example to illustrate the data flow after the image is inputted to the network. The first branch uses a 3 × 3 convolutional kernel with a relatively small receptive field. The schematic structure of this branch is shown as Figure 1.

The single-branch network design is inspired by the Resnet network (Xie et al., 2017) and consists of three parts. The first part is the CNN *Head*, which includes the convolutional layer, (group normalization (GN) layer, ReLu activation layer, and max pooling layer sequentially. The second and third parts are the two layers with similar structure, each containing two blocks. Through two layers, the feature maps will be inputted to the global average pooling layer and then be flattened into a one-dimensional feature vector.

The convolutional layer of CNN *Head* uses 64 convolution kernels of size 3 × 3 with depth 3 to filter the image of size 512 × 512 × 3. The stride is 2 pixels and the padding is 3 pixels. Then, the image goes through the GN layer and ReLu activation layer. Then, max pooling is performed on the feature maps. The size of pooling is 3 × 3, the stride is 2 pixels, and the padding is 1 pixel.

The basic architecture of the block in *Layer* is shown as follows.

**Figure 2:**
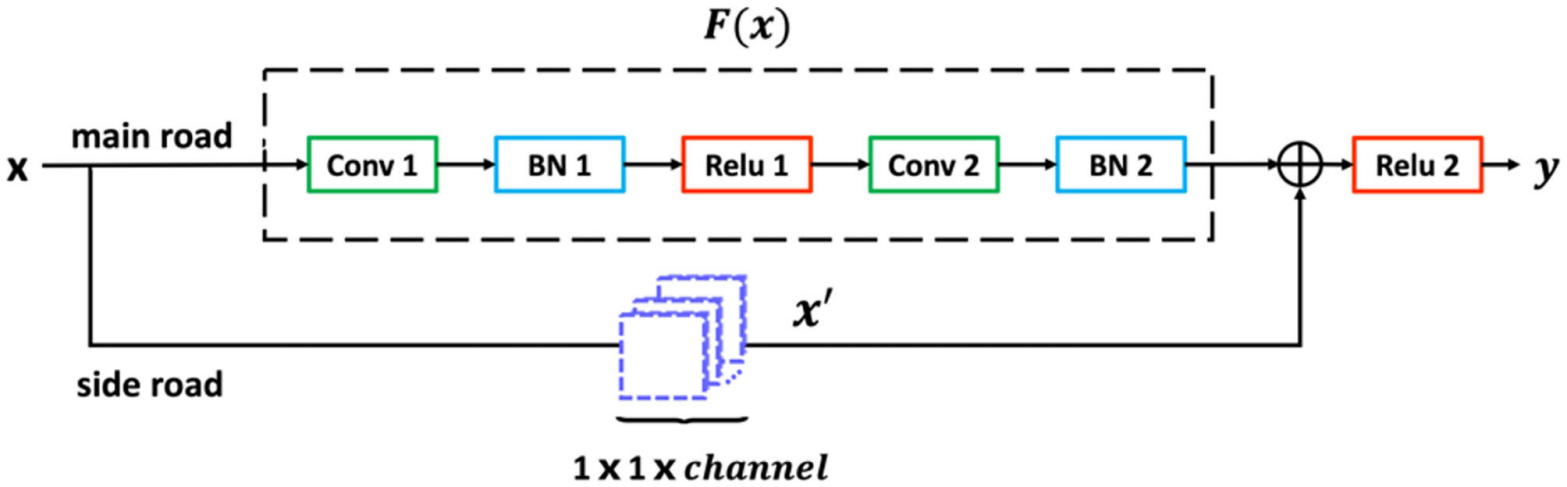
Architecture of *Block*.

A formula can express the output of the block

(1) $$\begin{array}{r} y\;=\;ReLu\left(F\left(x\right)\;+\;x^{\prime }\right) \end{array}$$

where *x* and *y* are, respectively, the input and output of the block. *F*(*x*) is the residual mapping which the network needs to learn, and *x*′ is the output from the side road. As shown in Figure 2, the block contains two roads, the main road and the side road. After entering the block, *x* will be inputted to the main road and the side road for data processing, respectively. In the main road, feature map *x* goes through the first convolution layer, the first batch normalization (BN) layer, the first ReLu activation layer, the second convolution layer, and the second BN layer and then becomes feature map *F*(*x*). To complete the summation in Eqn (1), the dimensions of *x* and *F*(*x*) must be equal. In the s side road, if the dimensions of *F*(*x*) are the same of *x*, no further data processing will be performed, which is called *x*′ = *x*. However, if the dimensions of the feature map output by the main road decrease, x needs to be processed by 1 × 1 convolution layer. The stride of 1 × 1 convolution kernel in Figure 2 is 2 pixels, and the padding is 1 pixel. The number of channels of the 1 × 1 convolutional layer depends on the number of channels outputting *F*(*x*) from the main road in the block. *F*(*x*) from the main road and *x*′ from the side road are activated by ReLu function after the summation of the pixels at the corresponding positions. The feature map y is the final output of this block.

There are two cascaded blocks in each layer with the same structure. The difference between the two blocks is that the resolution of the output *F*(*x*) in *Block1* decreases and the number of channels changes. In *Block2*, the stride of the first convolution layer is 1 pixel. Therefore, the size of feature map and the number of channels do not change, and a 1 × 1 convolution operation is not required in *Block2*. *Block1* uses a convolution kernel with the stride of 2 pixels instead of a pooling layer to reduce the resolution of the feature map. Springenberg et al. (2014) show that max pooling layer can be replaced by a convolution layer with a stride bigger than 1 pixel without loss of accuracy. Moreover, this can reduce the error to some degree in vast majority of networks. Therefore, we use stride of 2 pixels to compress the size of the feature map to ensure that the network not only reduces the dimension and removes redundant information, but also improves the discriminative accuracy.

*Layer2* has nearly the same design as of *Layer1*, except that *Layer1* has 128 convolutional channels and *Layer2* has 64 convolutional channels. Moreover, when the image is put into the single-branch benchmark network, it passes through the CNN *Head* and 2 layers one by one. Then, the network uses global average pooling to further reduce the resolution of the image and flatten the high-dimensional feature map into a one-dimensional feature vector, pending further classification processing.

Based on the above single-branch architecture network of *Branch1*, other two branches with similar structures can be designed, which are *Branch2* and *Branch3*. The main difference is that the size of the convolutional kernel used in *Branch2* is 5 × 5, and the size of the convolutional kernel used in *Branch3* is 7 × 7. Moreover, to make the feature map of each branch consistent in size for comparison and calculation, the padding of different branches is slightly different. *Branch2* uses 5 × 5 convolutional kernels, and the padding of the first block in each layer is 2 pixels. *Branch3* uses 7 × 7 convolutional kernels, the padding of the *head* convolutional layer is 4, and the padding of the first block in each layer is 3 pixels.

#### Overall Architecture of SMS-PCNN Model

Generally, ship images are natural scene images, so the input of SMS-PCNN model is RGB images. Analyzing the ship dataset, we found that the difference in appearance between different categories of ships mainly lies in the hull structure of ships, while the difference in appearance between ships of the same category lies in the superstructures and equipment of ships.

Therefore, for different categories of ships, we should pay more attention to discriminating architectural differences and use convolutional kernels with large receptive fields, which are more conducive to extracting features such as hull structures that occupy large pixel areas. For light differences between ships of the same category, we should use convolutional kernels with small receptive fields to extract features such as ship equipment and superstructures that occupy small pixel areas. For example, there are huge differences between the hull of the Ariake class destroyer and landing ship, which are two different categories of ships, so the convolution kernel with large receptive field is more advantageous. While aircraft carriers contain many categories of ships such as Queen Elizabeth class, the Nimitz class, the main difference between them is the bridge and other special equipment that occupy small pixel areas. In this case, the convolution kernel with smaller receptive field is more advantageous.

**Figure 3:**
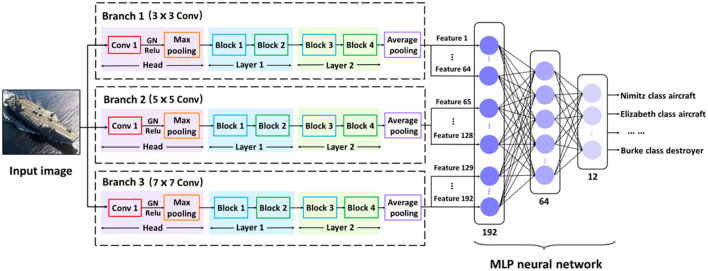
SMS-PCNN model.

Combining the above two considerations, the SMS-PCNN model uses different sizes of receptive fields to extract features of ship images at different levels, so as to achieve a high classification accuracy. The SMS-PCNN model has three parallel branches with different sizes of convolutional kernels that is 3 × 3, 5 × 5, and 7 × 7. The receptive fields of the branches increase, so that the features of the ship images can be extracted and classified in a multi-scale manner. The network structure of SMS-PCNN model is shown in the Figure 3.

The number of channels in the SMS-PCNN model has been adjusted two times. In the CNN *Head*, the convolution layer contains 64 channels. In *Layer1*, this number is expanded to 128. The reason is that the feature map shrinks and part of information loses during the process of extracting features from low-dimensional to high-dimensional features. To expand the amount of data flow between layers as much as possible, we expand the number of channels. However, the number of channels is reduced to 64 in *Layer2*. That is because the redundant information increases in the process of further abstraction of high-dimensional features. So, as to further improve the accuracy and reduce the redundant feature information, we decrease the number of channels.

After generated from three parallel branches with different sizes of receptive fields, feature vectors are linearly concatenated in the SMS-PCNN model.

(2) $$\begin{array}{r} {Fused\;\left(featurevector\right)}_{1\;\times \;p}\;=\;[{featurevector\left(branch1\right)}_{1\times q\;},\\ {featurevector\left(branch2\right)}_{1\times q\;},\;{feature\;vector\left(branch3\right)}_{1\;\times \;q\;}], \end{array}$$

where *p* and *q* denote the dimensions of feature vectors. *p* is 192 and *q* is 64. *Branch1* denotes the branch which uses 3 × 3 convolution, *Branch2* denotes the branch which uses 5 × 5 convolution, and *Branch3* denotes the branch which uses 7 × 7 convolution.

These fused feature vectors are processed by multi-layer perceptron neural networks (MLP neural networks), mapping feature vectors with different sizes of receptive fields into different classifications of ship images by decreasing the dimension layer by layer.

Suppose after flatten layer, MLP with *L* layer is used for feature classification, then the layer *l* has *m*^*l*^ neurons and the input vector is

(3) $$\begin{array}{r} a^{l\;-\;1}\;=\;{\;\left[a_{1}^{l\;-\;1},\;a_{2}^{l\;-\;1},\ldots ,a_{m^{l}}^{l\;-\;1}\right]}^{T} \end{array}$$

*a*^*l*−1^ is called the feature vector from layer (*l* − 1). The matrix of weight connected between neurons in layer (*l* − 1) and *l* is:

(4) $$\begin{array}{r} W^{l}\;=\;\left[\begin{array}{cc} \begin{array}{c} w_{11}^{l}\\ w_{21}^{l} \end{array} & \begin{array}{c} \begin{array}{ccc} w_{12}^{l} & \cdots & w_{1m^{l-1}}^{l} \end{array}\\ \begin{array}{ccc} w_{22}^{l} & \cdots & w_{2m^{l-1}}^{l} \end{array} \end{array}\\ \begin{array}{c} \vdots \\ w_{m^{l}1}^{l} \end{array} & \begin{array}{ccc} \begin{array}{c} \vdots \\ w_{m^{l}2}^{l} \end{array} & \begin{array}{c} \ddots \\ \cdots \end{array} & \begin{array}{c} \vdots \\ w_{m^{l}m^{l-1}}^{l} \end{array} \end{array} \end{array}\right] \end{array}$$

where $w_{jk}^{l}$ denotes the weights connected between neuron *k* in layer (*l* − 1) and neuron *j* in layer *l*.

The bias vector of neurons in layer *l* is

(5) $$\begin{array}{r} b^{l}\;=\;{\;\left[b_{1}^{l},b_{2}^{l},\ldots ,b_{m^{l}}^{l}\right]}^{T} \end{array}$$

then the feature vector *a*^*l*^ from neurons in layer *l* is

(6) $$\begin{array}{r} a^{l}\;=\;f^{l}\left(W^{l}•a^{l\;-\;1}\;+\;b^{l}\right) \end{array}$$

where the *f*^*l*^( ) is the activation function used by neurons in layer *l*. After the signal enters the feedforward neuron network, it passes layer by layer and gets the output *a*^*L*^ in the end. The whole process in the feedforward neural network is actually inputting the feature vectors from PCNN into the composite function *F*(.; *W, b*). Finally, the output of the network *a*^*L*^ is as follows.

(7) $$\begin{array}{r} a^{L}\;=\;F\left(x;W,b\right)\\ =\;f^{L}(W^{L}•f^{L-1}\left(\ldots W^{2}•f^{1}\left(W^{1}•x\;+\;b^{1}\right)\;+\;b^{2}\ldots \right)\\ +\;b^{L}) \end{array}$$

After the multilayer perceptron neural network, the SMS-PCNN model uses softmax function to normalize the vector *a*^*L*^. Let the i-th element of the vector *a*^*L*^ be $a_{i}^{L}$, then the result obtained after $a_{i}^{L}$ being processed by softmax function is

(8) $$\begin{array}{r} S_{i}\;=\;softmax\left(a_{i}^{L}\right)\;=\;\frac{\exp\left(a_{i}^{L}\right)}{\sum_{j}\exp\left(a_{j}^{L}\right)} \end{array}$$

After softmax function, $a_{i}^{L}$ is mapped to the *S*_*i*_.*S*_*i*_ satisfies the properties of the probability distribution, *S*_*i*_ ∈ (0, 1) and $\underset{i}{\sum }S_{i}=1$. Therefore, *S*_*i*_ can represent the prediction probability. By selecting the node with the largest prediction probability, we can obtain the final classification category.

The predicted probability *S*_*i*_ will also be inputted to loss function. In the SMS-PCNN model, we use cross-entropy loss function used. It is

(9) $$\begin{array}{r} Loss\;=\;-\overset{K}{\underset{i\;=\;1}{\sum }}p_{i}\log S_{i} \end{array}$$

where *K* is the number of categories, and *p*_*i*_ denotes the indicator variable (takes the value of 0 or 1). If the predicted category of the sample *i* is the same as its true category, *p*_*i*_ takes the value of 1 and 0 otherwise. Cross-entropy loss function can measure the prediction performance of the model.

To address the problem of too many parameters in the SMS-PCNN model, the SMS-PCNN model uses L2 regularization to mitigate overfitting. Regularization is a method to reduce the complexity of the model by introducing penalty terms. The L2 regularization is to add the L2 norm of the parameters after the loss function:

(10) $$\begin{array}{r} \hat{L}oss\;=\;-\overset{K}{\underset{i\;=\;1}{\sum }}p_{i}\log S_{i}\;+\;\alpha \Omega \left(w\right) \end{array}$$

where $\hat{L}oss$ is cross-entropy loss function with L2 regularization, and *w* is the weight coefficient matrix, and the parameter α controls the strength of regularization. Ω function is the L2 norm, namely, the sum of squares of the weight coefficients:

(11) $$\begin{array}{r} \Omega \left(w\right)\;=\;||w|{|}_{2}^{2} \end{array}$$

### Tricks for Model Optimization

#### Optimization for Specific Ship Dataset Applications

Generally, the distinguishing features of some ships are mainly concentrated in parts that account for a small proportion of the images, such as the bridge of aircraft carriers and the radar of frigates. Therefore, to improve the accuracy of the model, we need to retain as much information of images as possible and use images with large size to contain more information as much as possible. For images with large size, the batch size is reduced to fit the training. However, relatively low batch size will lead to bad performance of the batch normalization (BN) used in the SMS-PCNN model. In this case, Wu and Kaiming (2020) proposed group normalization (GN) to improve the dependence of BN on batch size and proved that when the batch size is <8, the error of using GN is smaller than BN.

The GN layer calculates the mean and variance based on the input data and then uses these two values to normalize the input data. Specifically, GN divides the channels of the layer into groups and uses the mean and variance of the data within the groups.

(12) $$\begin{array}{r} \hat{x_{i}}\;=\;\frac{1}{\sigma_{i}}\left(x_{i}\;-\;\mu_{i}\right) \end{array}$$

$\hat{x_{i}}$ is the feature map of the layer, and *i* = (*i*_*N*_, *i*_*C*_, *i*_*H*_, *i*_*w*_). *N* is the batch axis, *C* is the channel axis, *H* and *W* are the spatial height and width axis. μ and σ here can be calculated by the following equations

(13) $$\begin{array}{r} \mu_{i}\;=\;\frac{1}{m}\underset{k\in S_{i}}{\sum }x_{k} \end{array}$$

(14) $$\begin{array}{r} \sigma_{i}=\sqrt{\frac{1}{m}\underset{k\in S_{i}}{\sum }{\left(x_{k}\;-\;\mu_{i}\right)}^{2}\;+\;\epsilon } \end{array}$$

where ε is a small constant. *S*_*i*_ is a feature collection with a size of *m* used to calculate μ and σ. The solution of *S*_*i*_ in GN is the following equation.

(15) $$\begin{array}{r} S_{i}\;=\;\left\{k|k_{N}\;=\;i_{N},\left\lfloor \frac{k_{C}}{\frac{C}{G}}\right\rfloor \;=\;\left\lfloor \frac{i_{C}}{\frac{C}{G}}\right\rfloor \right\} \end{array}$$

*G* is the number of groups. In the SMS-PCNN model, we pre-set *G* = 2. *C* is the number of channels, and *C/G* is the number of channels in each group. “$\left\lfloor \frac{k_{C}}{\frac{C}{G}}\right\rfloor =\left\lfloor \frac{i_{C}}{\frac{C}{G}}\right\rfloor$” indicates that *i* and *k* are in the same channel group.

When we used GN in all the normalization layers of the SMS-PCNN models, gradient explosion appeared. Therefore, we only use GN to normalize the output of the head convolution layer of each branch whereas BN is used in other layers, which achieved good results.

#### Learning Rate Optimization

The model uses gradient descent to minimize the value of loss function and thus converge to the optimal solution. During the iterations performed by the gradient descent, the learning rate controls the learning progress of the model. To optimize the model learning rate, we use cosine annealing and Warmup (He et al., 2016) technique. Warmup is used to train with a small learning rate at the beginning of to make the network familiar with the data. As the training continues, the learning rate slowly increases and reaches the initial learning rate within a pre-set iteration. Then, we use cosine annealing to adjust learning rate.

The equation of decreasing learning rate is as follows:

(16) $$\begin{array}{r} \eta_{t}\;=\;\eta_{\min}\;+\;\frac{1}{2}\left(\eta_{\max}\;-\;\eta_{\min}\right)\left(1\;+\;\cos\left(\frac{T_{cur}}{T}\pi \right)\right) \end{array}$$

where η_*t*_ is the learning rate of the *t*-th iteration. η_min_ is the termination learning rate. η_max_ is the initial learning rate. *T*_*cur*_ is the current iteration. *T* is the total iteration. The key of the formula is $\cos\left(\frac{T_{cur}}{T}\pi \right)$. As iteration increases, the value of $\cos\left(\frac{T_{cur}}{T}\pi \right)$ decreases from 1 to −1, so that the learning rate η_max_ decreases to η_min_.

**Figure 4:**
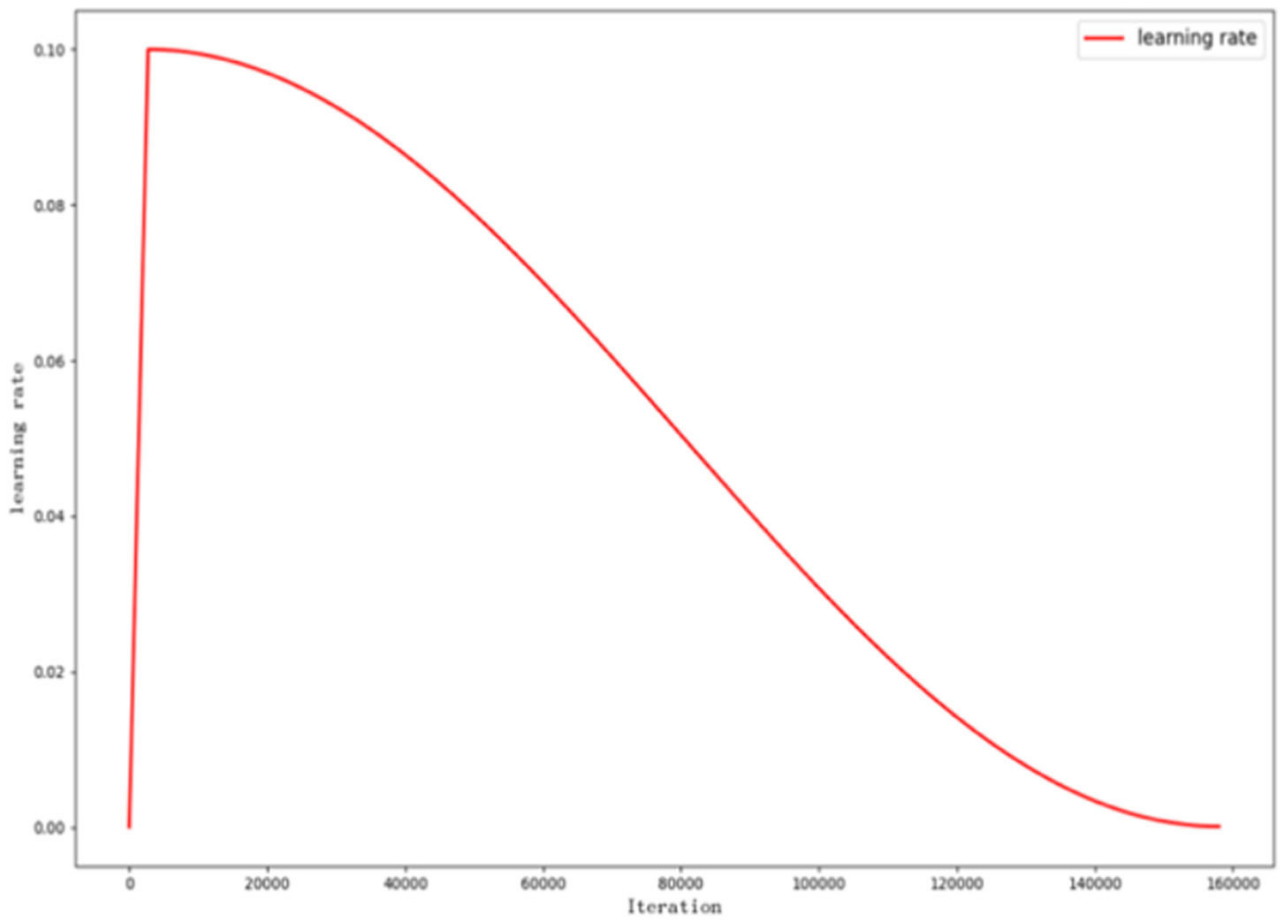
Curve of learning rate adjustment.

The learning rate adjustment for the whole model training is shown in the Figure 4.

The learning rate is adjusted with iterations. In the first 2,709 iterations (3 epochs), warmup is used. As iteration gradually increases, the learning rate grows to 0.1, and then, this number decreases to 10^−4^ using cosine annealing.

#### Optimization of Overfitting of the Ship Dataset

On the Internet, ship images are limited in amounts and in low quality, so the ship dataset we compiled from open-source data is limited in size, which leads to overfitting to some degrees when we use the SMS-PCNN model for training. For this reason, we use two methods, label smoothing and dropout, to mitigate overfitting in training.

Label smoothing is a technique proposed by Sergey and Christian (2015) in the model of inception-V2 to mitigate overfitting. The traditional one-hot coding suffers from overconfidence, which leads to overfitting. To address overconfidence, label smoothing decays the item with probability 1 in one-hot, which reduces the weight of category of the ground-truth label in the calculation of loss function. The confidence of the decayed items in one-hot is equally divided into other items, so that each category has a certain amount of confidence, which can suppress overfitting. In the SMS-PCNN model, we add the label smoothing to cross-entropy loss function, and loss function is changed as follows.

(17) $$\begin{array}{r} Loss\left(p,q\right)\;=\;-\overset{K}{\underset{i\;=\;1}{\sum }}p_{i}\log q_{i}\to \\ \hat{L}oss\left(p^{\prime },q\right)\;=\;-\overset{K}{\underset{i\;=\;1}{\sum }}p_{i}^{\prime }\log q_{i} \end{array}$$

where *K* denotes the number of categories, and *p*_*i*_ denotes the indicator variable (takes the value of 0 or 1). *q*_*i*_ is the predicted probability value.

(18) $$\begin{array}{r} p_{i}^{\prime }\;=\;\left(1\;-\;\varepsilon \right)p_{i}\;+\;\varepsilon u\left(k\right) \end{array}$$

where ε is a smoothing parameter, which is a pre-set hyperparameter. *u*(*k*) is a probability distribution and uniform distribution is used here, so $u\left(k\right)=\frac{1}{K}$. The result of cross-entropy loss function through label smoothing $\hat{L}oss\left(p^{\prime },q\right)$ is as follows.

(19) $$\begin{array}{r} \hat{L}oss\left(p^{\prime },q\right)\;=\;(1\;-\;\varepsilon \;Loss\left(p,q\right)\;+\;\varepsilon \;Loss\left(u,q\right) \end{array}$$

In addition to label smoothing, we also use dropout to prevent the model from overfitting. In dropout (Hou et al., 2019), the training process randomly drops out some neurons in hidden layers and maintains the number of neurons in input and output layers. Then, we inputted data through the modified network to propagate forward, and the loss which is calculated by the network is propagated back. Suppose the multilayer perceptron neural network has L layers, and *l* ∈ {1 …, *L*} denotes the neuron in layer *l*. Let *z*^*l*^ denotes the input of the neuron in layer *l*, *y*^*l*^ denotes the output of the neuron in layer *l*, and *W*^*l*^ and *b*^*l*^ denote the weight coefficients and bias of the neuron in layer *l*. Then, the feedforward neural network can be expressed by the following equation.

(20) $$\begin{array}{r} z^{\left(l\;+\;1\right)}\;=\;W^{\left(l\;+\;1\right)}y^{l}\;+\;b^{\left(l\;+\;1\right)} \end{array}$$

(21) $$\begin{array}{r} y^{\left(l\;+\;1\right)}\;=\;f\left(z^{\left(l+1\right)}\right) \end{array}$$

where *f* denotes the activation function. When we use dropout during the model training, the feedforward neural network becomes of the following form.

(22) $$\begin{array}{r} r_{i}^{l}\;\sim \;Bernoulli\left(p\right) \end{array}$$

(23) $$\begin{array}{r} {ỹ}^{\left(l\right)}\;=\;r^{\left(l\right)}\;*\;y^{\left(l\right)} \end{array}$$

(24) $$\begin{array}{r} z^{\left(l+1\right)}\;=\;W^{\left(l\;+\;1\right)}{ỹ}^{l}\;+\;b^{\left(l\;+\;1\right)} \end{array}$$

(25) $$\begin{array}{r} y^{\left(l\;+\;1\right)}\;=\;f\left(z^{\left(l\;+\;1\right)}\right) \end{array}$$

where the probability *p is a* pre-set hyperparameter. *r*^*l*^ is a random variable that obeys the Bernoulli distribution and is designed to randomly generate a vector of 0 or 1 with probability *p*. Probability *p* denotes the proportion of the elements of this vector taking the value of 0. *r*^*l*^ is to sample each layer and multiply *y*^(*l*)^ which is the output of that layer element by element. *y*^(*l*+1)^ denotes the final result of Dropout.

During the prediction of the model, the weight parameter of each neural unit is multiplied by the probability *p*, $w_{test}^{\left(l\right)}\;=\;pW^{\left(l\right)}$.

In the SMS-PCNN model, we used the dropout before two fully connected layers and set the probability *p* to be 0.3, which means that 30% of the neurons were dropped out randomly, and the experiment achieved a good accuracy improvement.

## Experiment

### Dataset

**Figure 5:**
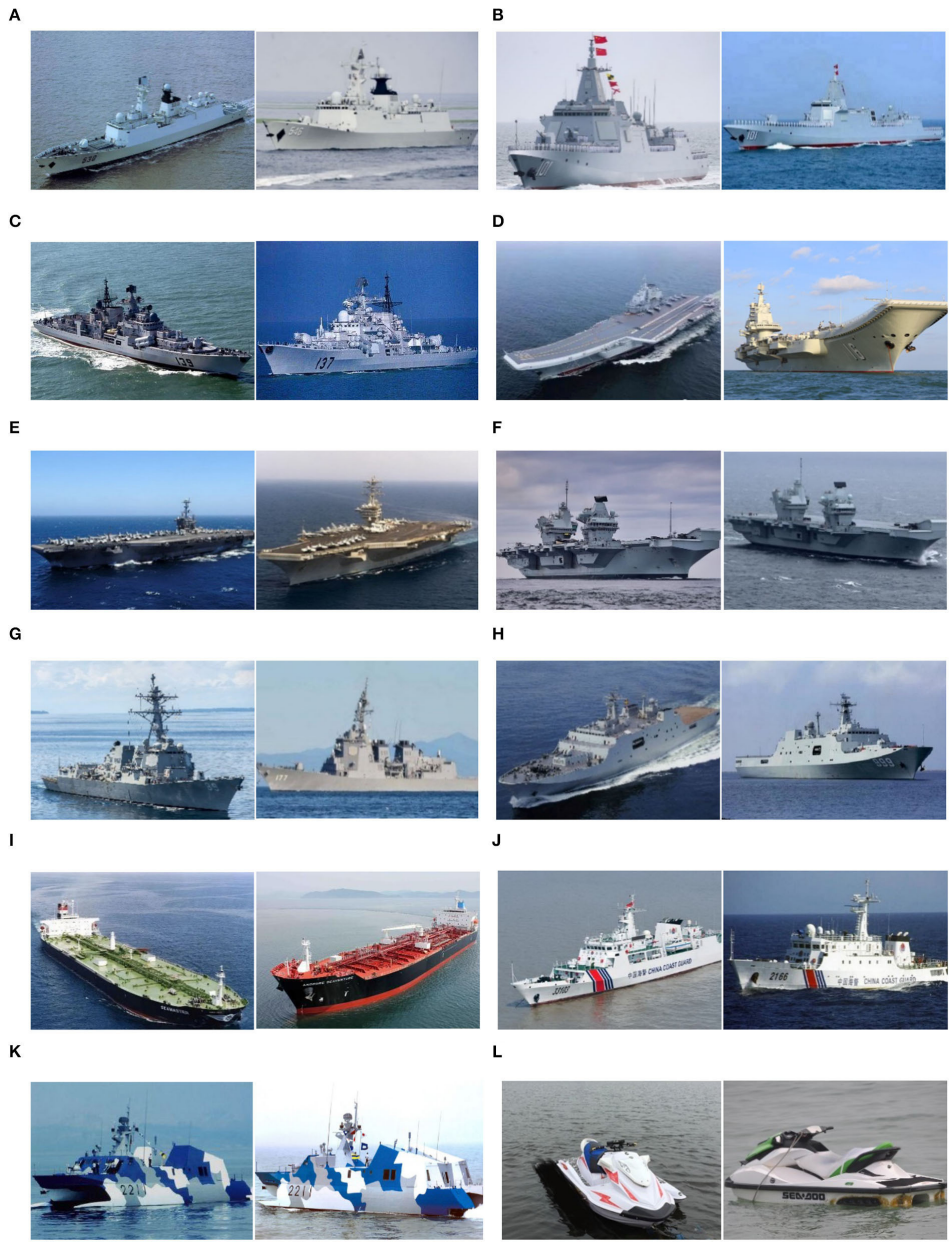
Ship dataset. **(A)** Type 054 frigate, **(B)** Type 055 destroyer, **(C)** Sovremenny class destroyer, **(D)** Chinese aircraft carrier Liaoning, **(E)** Nimitz class aircraft carrier, **(F)** Queen Elizabeth-class aircraft carrier, **(G)** Arleigh Burke class destroyer, **(H)** Landing ship, **(I)** Oil tanker, **(J)** Surveillance boat, **(K)** Missile boat, and **(L)** Motorboat.

On the Internet, ship images are limited in amounts and in low quality. Therefore, there are no large-scale standard open-source ship datasets. We collected open-source images from the Internet and compiled a dataset containing 12 categories of ship targets. Part of the ship images is shown in Figure 5.

The number of each sample of 12 categories applicable to the image classification task is shown in Table 1.

**Table 1:** Distribution of ship dataset.

**Ship Category**	**Number of images**	**Ship category**	**Number of images**
Surveillance boat	397	Type 055 destroyer	409
Motorboat	474	Arleigh Burke class destroyer	445
Landing ship	495	Type 054 frigate	404
Missile boat	403	Chinese aircraft carrier Liaoning	378
Oil tanker	436	Nimitz-class aircraft carriers	502
Sovremenny class destroyer	420	Queen Elizabeth-class aircraft carriers	299

In the ship dataset shown in Table 1, we divide the training set and valid set according to the ratio of 7:3.

### Experiment and Result

#### Experiment and Result of SMS-PCNN Model

We use the SMS-PCNN model optimized by three tricks to conduct experiments. Samples of 12 categories were trained iteratively using 175 epochs with the batch size of 4. The model was trained iteratively using the stochastic gradient descent (Sebastian, 2016). Cosine annealing and warmup optimization techniques were used on the learning rate. After warmup, the learning rate is initially set to 0.1 and finally decreased to 10^−4^. The weight of label smoothing is set to 10^−3^. The SMS-PCNN model uses L2 regularization and its weight is set to 10^−3^. MLP uses two dropouts, both set to discarding 30% of the neurons. The batch normalization, group normalization, and MLP in the model all use the correlation functions that come with PyTorch. After being preprocessed, the natural scene images with the adjusted resolution of 512 × 512 are inputted to the SMS-PCNN model for training.

Table 2 shows the architecture of SMS-PCNN model for a ship dataset with resolution of 512 × 512. Among them, the images are processed by *Branch1, Branch2*, and *Branch3* in parallel and then jointly processed by average pooling. Finally, the feature vectors from three branches are concatenated together. The concatenated feature vectors with 192 dimensions sequentially go through the input layer, the hidden layer with 64 neurons and the output layer with 12 neurons of the MLP neural network. Then, these vectors pass through the softmax function, and the results of classification are outputted.

**Table 2:** Architectures of SMS-PCNN model.

**Layer name**	**Output size**	**Branch1**	**Branch2**	**Branch3**
Conv1	256 × 256	3 × 3, 64, stride = 2 padding = 3	5 × 5, 64, stride = 2 padding = 3	7 × 7, 64, stride = 2 padding = 4
Pooling	129 × 129	3 × 3 max pooling, stride = 2, padding = 1		
Layer1	65 × 65	$\left(\begin{array}{c} 3*3,128\\ 3*3,128 \end{array}\right)\times 2,\;\mathrm{padding}=1$	$\left(\begin{array}{c} 5*5,128\\ 5*5,128 \end{array}\right)\times 2,\;\mathrm{padding}=2$	$\left(\begin{array}{c} 5*5,128\\ 5*5,128 \end{array}\right)\times 2,\;\mathrm{padding}=2$
Layer2	33 × 33	$\left(\begin{array}{c} 3*3,128\\ 3*3,128 \end{array}\right)\times 2,\;\mathrm{padding}=1$	$\left(\begin{array}{c} 5*5,64\\ 5*5,64 \end{array}\right)\times 2,\;\mathrm{padding}=2$	$\left(\begin{array}{c} 5*5,64\\ 5*5,64 \end{array}\right)\times 2,\;\mathrm{padding}=3$
	1 × 1	Average pooling		
Fully connected layer1		(192, 64), dropout = 0.3		
Fully connected layer2		(64, 12), dropout = 0.3		

The “64” in Conv1 denotes the number of convolutional kernels. The “128” in *Layer1* and 64 in *Layer2* both denote the number of convolutional kernels. The “×2” of each Branch in *Layer1* and *Layer2* denotes that the two cascaded blocks using the same parameters. Take the parameters of fully connected layer1 (192, 64) as an example, “192” indicates the dimension of the input vector and “64” the dimension of the output vector.

Generally, the indicator to evaluate the classification effectiveness of a classifier is its accuracy. The classification accuracy is the proportion of correctly predicted results to the total sample.

**Figure 6:**
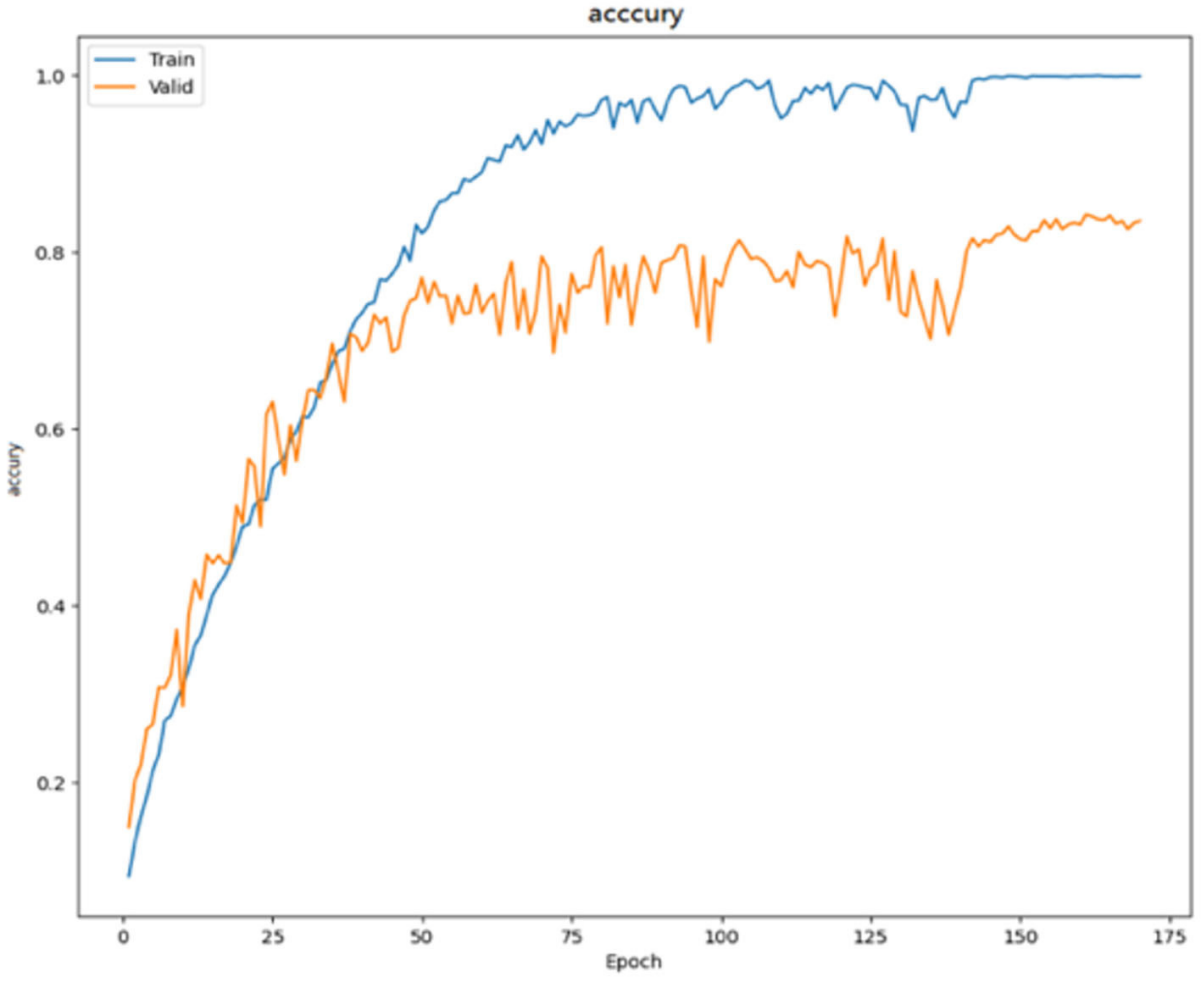
Accuracy curve.

As seen in Figure 6, the SMS-PCNN model, which combines all tricks, converges faster in training and has nearly reached the highest accuracy rate at the 100th epochs. With the increase of epochs, the learning rate gradually decreases and the training gradually tends to be stable, and the final model optimal results reach 84.79% accuracy.

**Figure 7:**
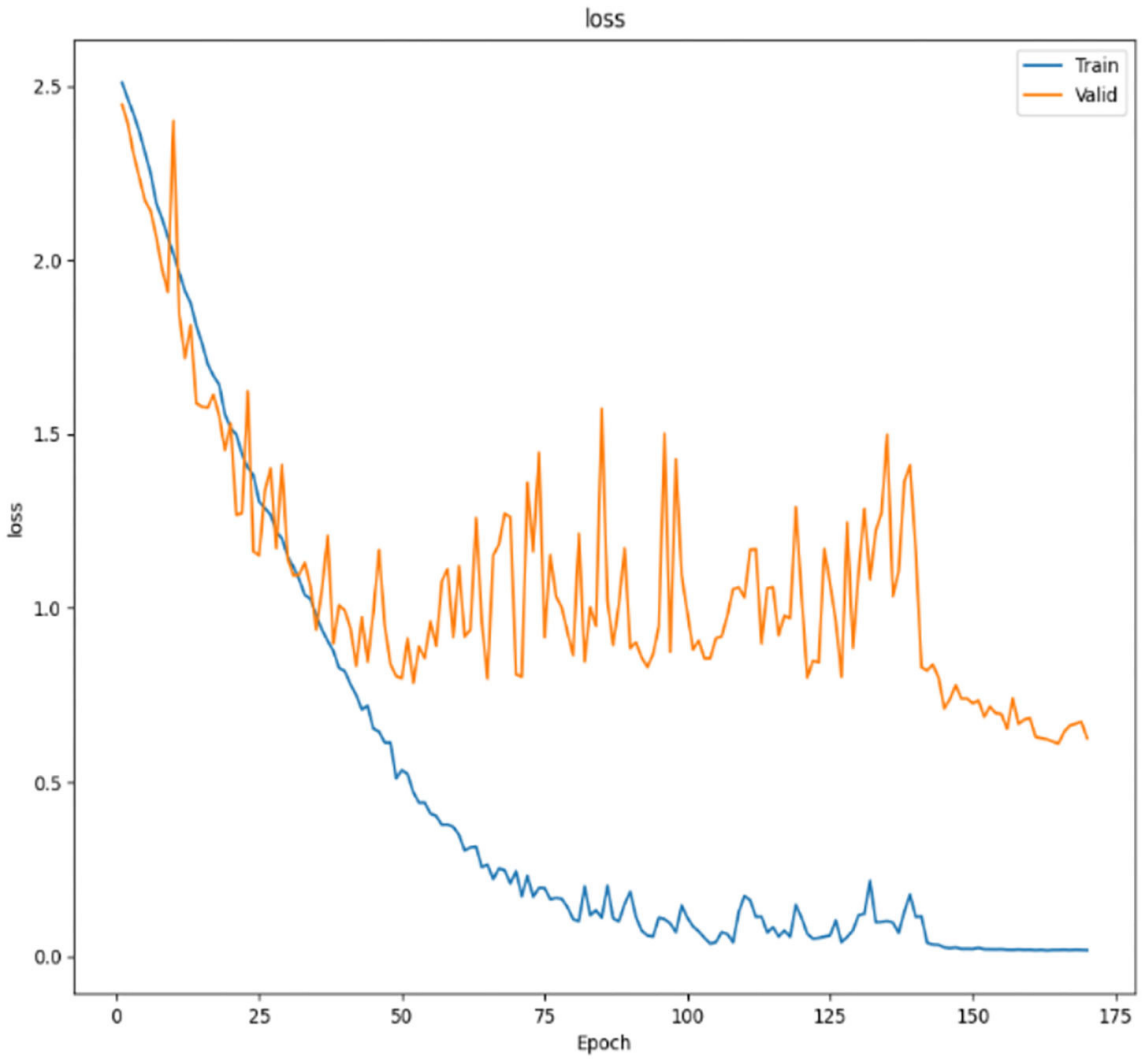
Loss curve.

The loss function in the SMS-PCNN model uses cross entropy loss function with label smoothing and is shown in the Figure 7. The improved loss function converges faster and more stably in the training set. At the end of the training, the model training finally converges. The decrease of the loss function on the valid set tends to be stable as the learning rate decreases and reaches the optimal result around 150th epochs. The confusion matrix of the SMS-PCNN model is shown in the following figure.

**Figure 8:**
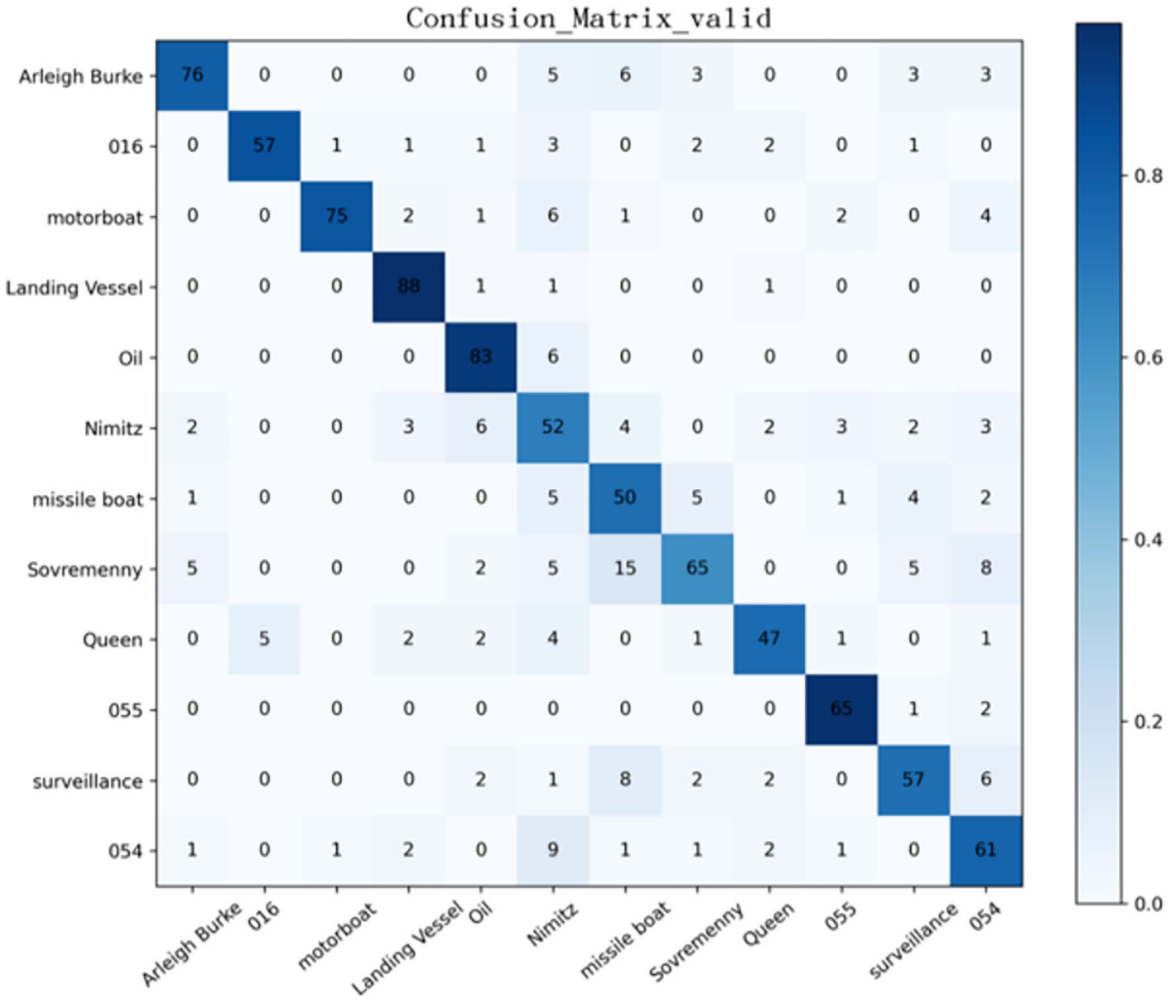
Confusion matrix of the valid set of SMS-PCNN model.

From the confusion matrix of the SMS-PCNN model on the valid set as shown in Figure 8, three points can be concluded. (1) The darkest areas of the confusion matrix are concentrated in the diagonal line, showing great classification performance. (2) Among all categories, landing ships, type 055 destroyers, and oil tankers have the highest classification accuracy. (3) Sovremenny class destroyers and Nimitz class aircraft carriers cannot be classified well. The reason why these two categories have a slightly worse classification performance than others is that there are diverse ships of these two categories with minor differences, resulting in the lack of obvious different features between categories.

**Figure 9:**
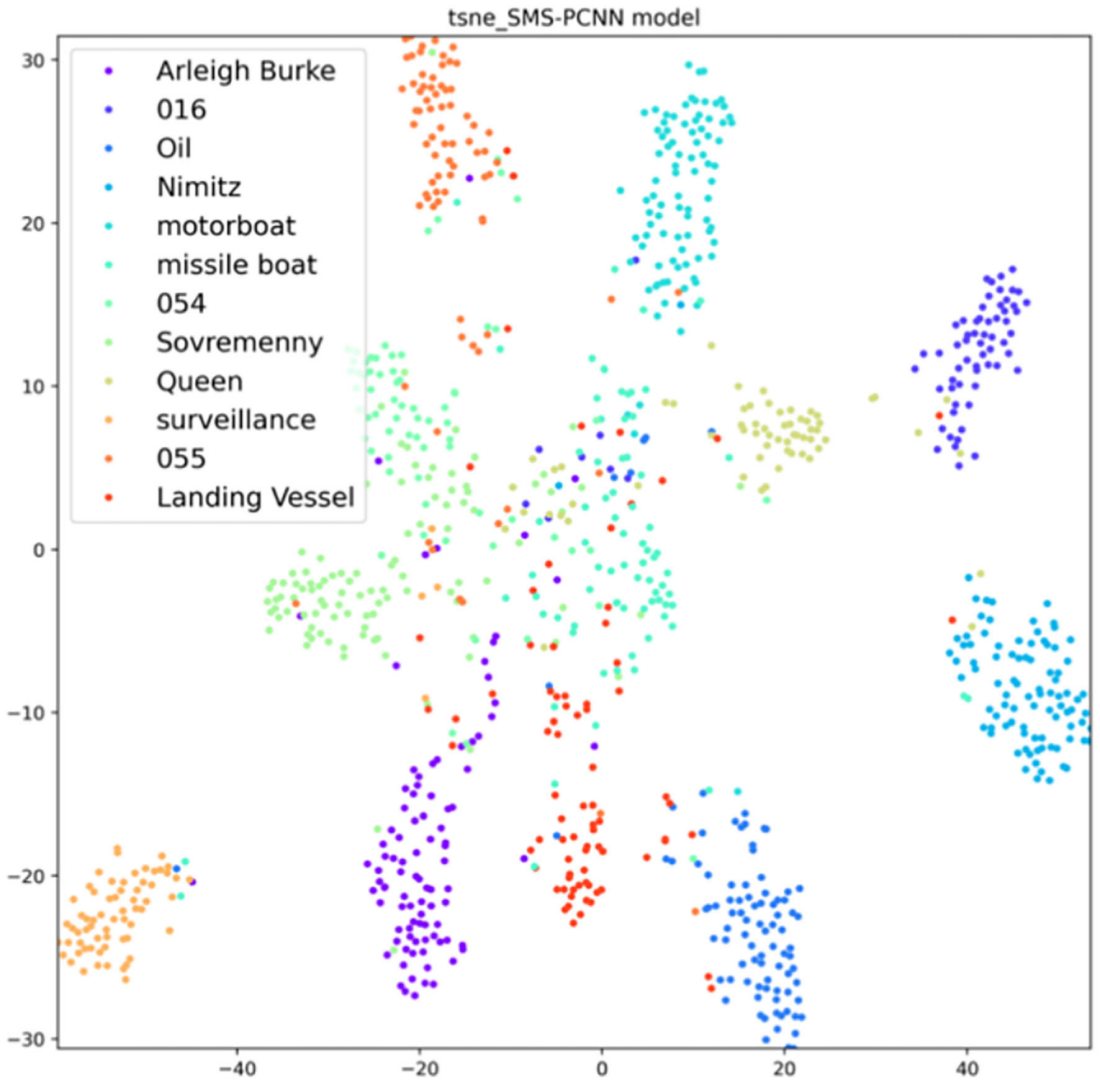
SMS-PCNN model t-sne clustering analysis.

T-sne (Laurens, 2014) clustering analysis of the SMS-PCNN model for the valid data is plotted in the Figure 9.

From the t-sne clustering analysis graph, we can find that the samples of each category are basically concentrated together, and the SMS-PCNN model can classify the samples well.

#### Performance Comparison

This section is divided into two parts for performance comparison. The first part is the performance comparison of the SMS-PCNN model with classical model frameworks and the model with single branch. The second part is the validation of the tricks of the SMS-PCNN model.

(1) Comparison to state-of-the-art approaches.

We compared our SMS-PCNN model with 4 state-of-the-art approaches: Resnet-50 (He et al., 2016), Alexnet (Alex et al., 2017), VGG-16 (Simonyan and Zisserman, 2014), and Resnet-18 (He et al., 2016). All the methods were compared on the valid set of our ship dataset. The accuracy comparison is presented in Table 3. The results in Table 3 show that the proposed SMS-PCNN model achieved the best results among all algorithms. All the state-of-the-art comparisons on the ship dataset are fine-tuned well. These comparisons use the same hyperparameters as the SMS-PCNN model we proposed.

**Table 3:** Accuracy comparison with state-of-the-art approaches.

**Model**	**Accuracy (%)**
Resnet-50	63.82
Alexnet	64.23
VGG-16	67.52
Resnet-18	70.09
Branch1-CNN (3 × 3 conv)	75.53
SMS-PCNN model	84.79

In addition, we conducted experiments on network architecture with single branch to analyze the classification of ships. The experiments showed that the Branch1-CNN with small receptive field of 3 × 3 convolutional kernel achieved an accuracy of 75.53%, which is lower than the accuracy of the SMS-PCNN model. This demonstrates the necessity of designing a multi-branch structure.

**Figure 10:**
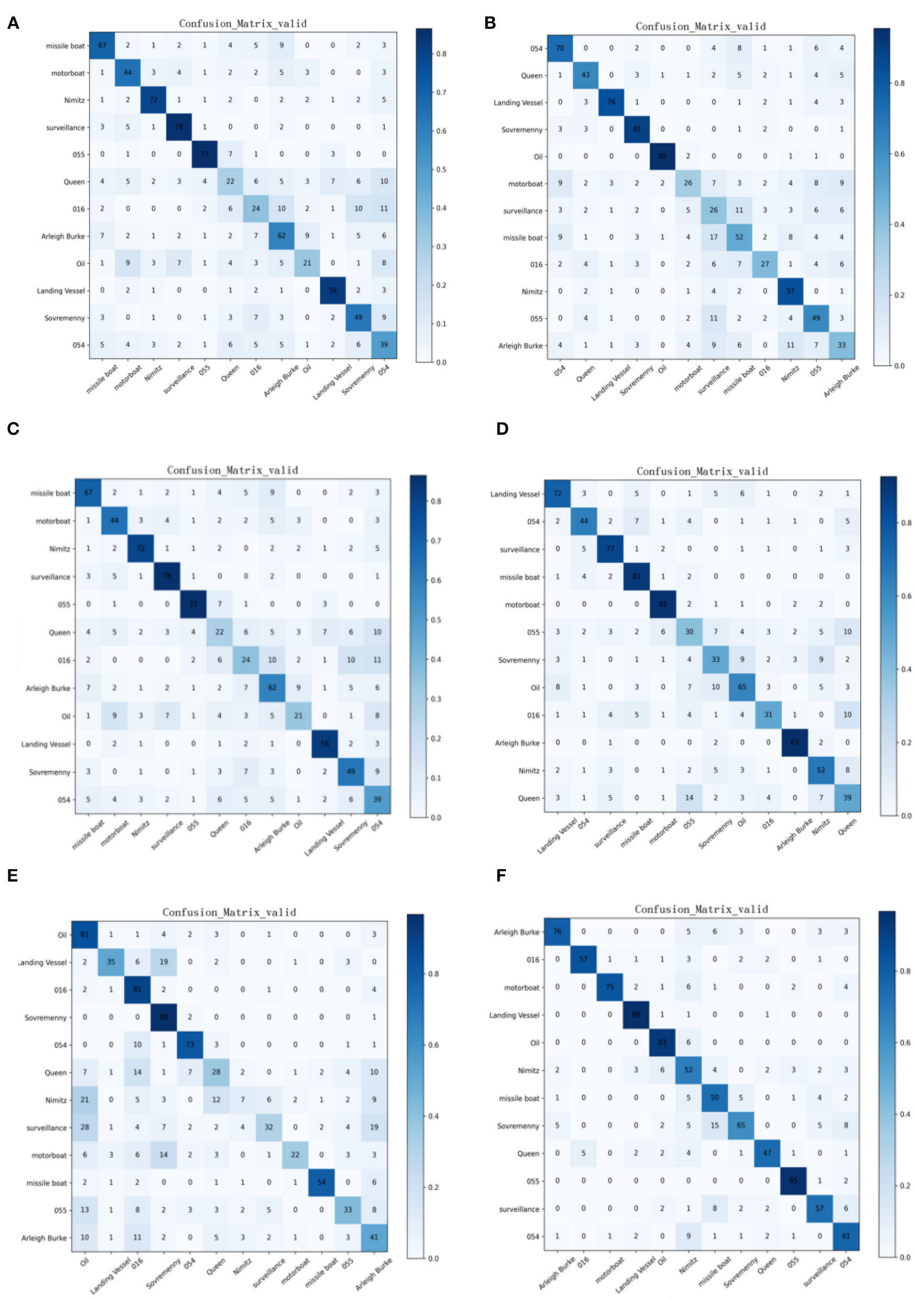
Confusion matrix of state-of-the-art approaches and Branch1-CNN in valid dataset. **(A)** Resnet-50, **(B)** Alexnet, **(C)** VGG-16, **(D)** Resnet-18, **(E)** Branch1-CNN (3 × 3 conv), **(F)** SMS-PCNN model.

**Figure 11:**
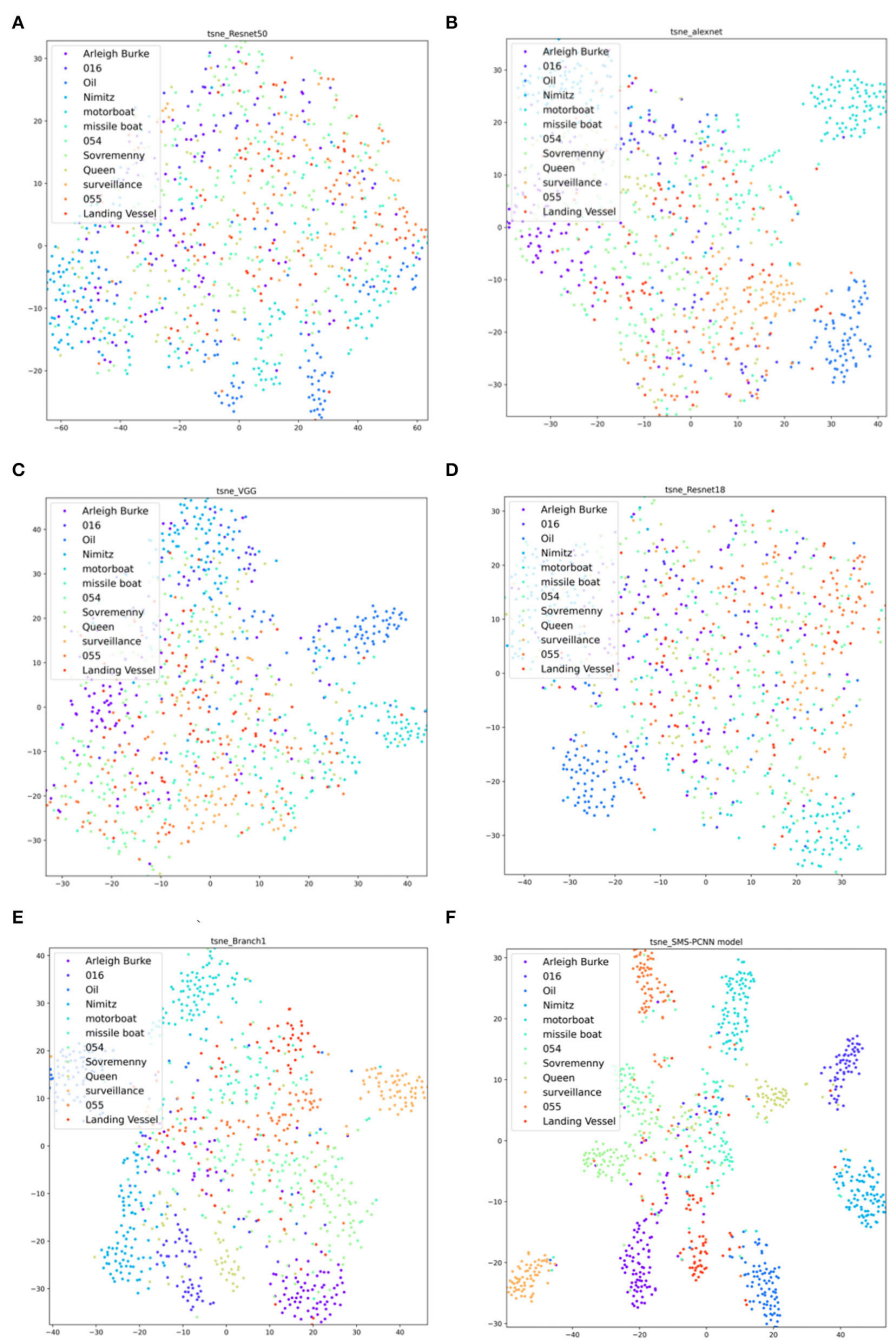
T-sne clustering analysis of state-of-the-art approaches in valid dataset. **(A)** Resnet-50, **(B)** Alexnet, **(C)** VGG-16, **(D)** Resnet-18, **(E)** Branch1-CNN (3 × 3 conv), **(F)** SMS-PCNN model.

Comparing with the confusion matrix and t-sne clustering analysis graph in Figures 10, 11, it can be clearly concluded that compared with the SMS-PCNN model, other models do not have well classification effect and have limitations in generalization performance. The confusion matrix shows that other models have low accuracy in discriminating the Queen Elizabeth-class aircraft carrier and the Chinese aircraft carrier Liaoning. The t-sne clustering analysis shows that the point representing Queen Elizabeth-class aircraft carrier and the Chinese aircraft carrier Liaoning are scattered and disorganized, showing a bad performance on clustering. The results of confusion matrix and t-sne clustering analysis are consistent and unified.

(2) Validation of tricks of SMS-PCNN model.

Basic SMS-PCNN means the SMS-PCNN model we used to perform an experiment without trick1, trick2, and trick3. These tricks are used for optimizing the network, including enhancing specific ship dataset applications, improving training learning rate and avoiding overfitting, respectively.

We designed the ablation experiments. The result is shown in Table 4.

**Table 4:** Ablation experiments of SMS-PCNN model.

**Model**	**Accuracy (%)**
Basic SMS-PCNN	77.28
Basic SMS-PCNN(add trick1)	79.44
Basic SMS-PCNN(add trick1 and trick2)	83.24
SMS-PCNN(add trick1-3)	84.79

It demonstrates that the accuracy of Basic SMS-PCNN model without any tricks is 77.28%, which is still higher than the other state-of-the-art approaches and Branch1-CNN in valid dataset mentioned above. This demonstrates the superiority of the model design principles and the basic framework structure. Meanwhile, the ablation experiments examined that the accuracy of the Basic SMS-PCNN model with trick1 increases by 2.16–79.44%. For the Basic SMS-PCNN (add trick1 and trick2), the accuracy increases by 5.96–83.79%. When using trick1, trick2, and trick3, the model accuracy increases by 7.51–84.79%. It can be seen that the use of three tricks did improve the accuracy of SMS-PCNN model and enhance the classification effect.

#### Mechanistic Analysis of the Network

To further analyze the mechanism of the SMS-PCNN model and verify the effectiveness of parallelizing multi-scale convolutional branch and adjusting the number of channels, we visualize the convolutional kernels and the feature maps of tanker image through each convolutional module. The head convolution layer of each Branch in the SMS-PCNN model is a convolutional kernel with depth 3 designed for three channels of RGB. When visualizing, we combine these convolutional kernels into single convolutional kernel with color. The visualization of the 64 combined convolutional kernels of the head convolutional layer of each branch of the SMS-PCNN model is shown in the following figure.

**Figure 12:**
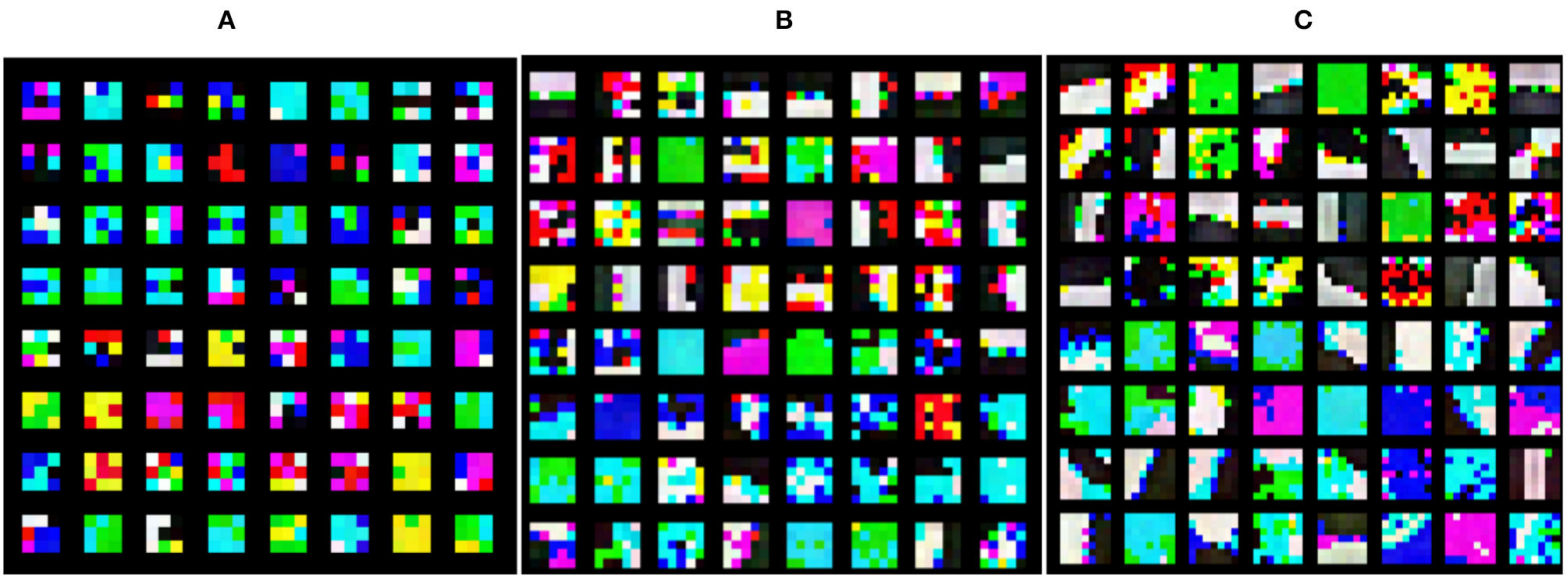
Convolutional kernel visualization. **(A)** Branch1_Head(3 × 3conv), **(B)** Branch2_Head(5 × 5 conv), **(C)** Branch3_Head(7 × 7 conv).

From the visualization of the convolution kernels in Figure 12, we can find that the head convolution layer of *Branch3* with 7 × 7 size convolution kernels mainly focuses on the edge features of images, so it can better extract features such as the hull of ships. The head convolution layers of *Branch2* and *Branch1* with 5 × 5 and 3 × 3 size convolution kernels focus on the fine-grained features of images, so it can better extract features such as ship superstructures and equipment.

We take the tanker image as an example to visualize feature maps that pass through each branch convolution module. In each branch, the tanker image passes through the CNN *Head, Layer1*, and *Layer2*, and the feature maps are shown in the following figure.

**Figure 13:**
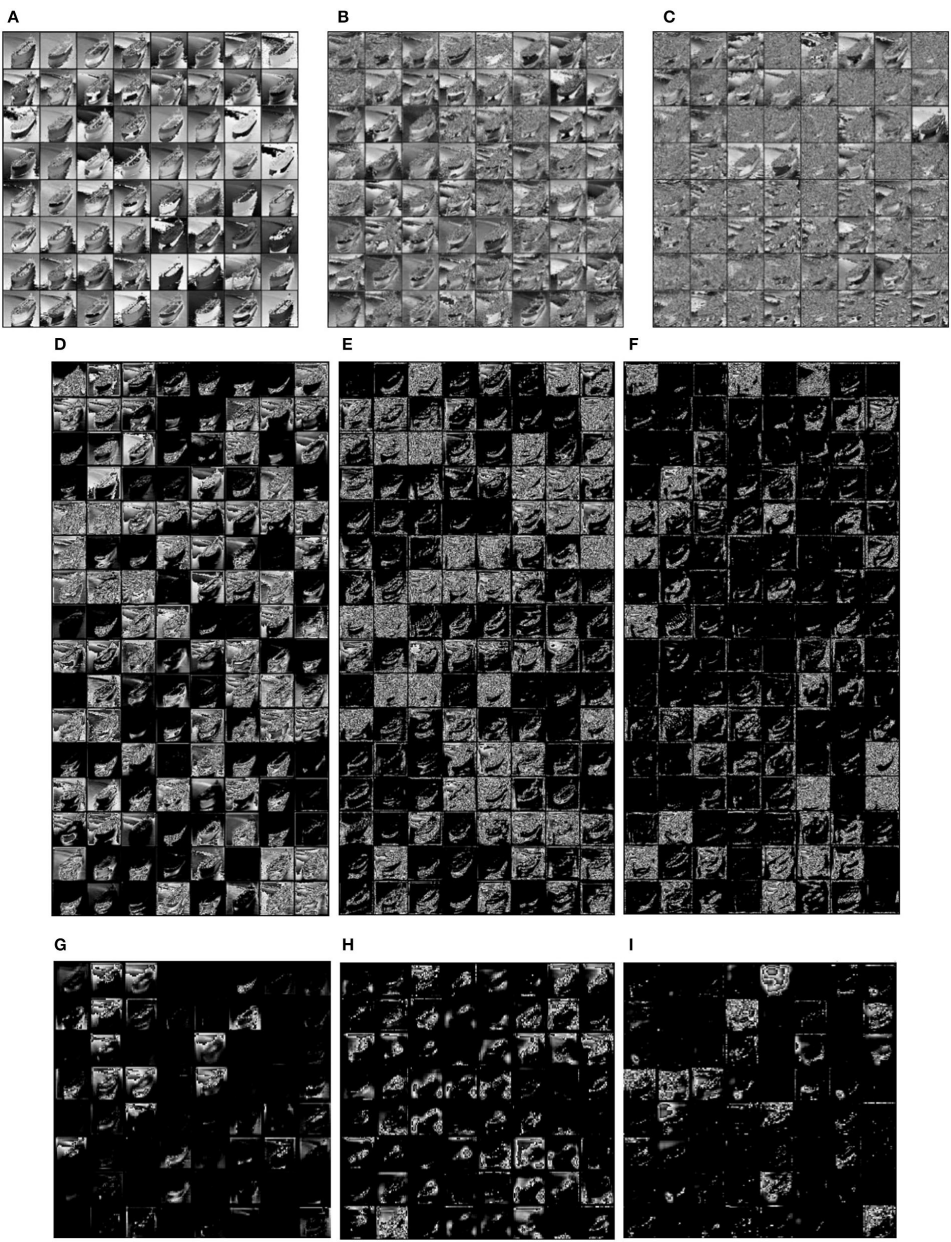
Feature maps of oil tanker image after different convolution layers. **(A)** Branch1_Head(3 × 3 conv), **(B)** Branch2_Head (5 × 5 conv), **(C)** Branch3_Head(7 × 7 conv), **(D)** Branch1_layer1 (3 × 3 conv), **(E)** Branch2_layer1 (5 × 5 conv), **(F)** Branch3_layer1(7 × 7 conv), **(G)** Branch1_layer2(3 × 3 conv), **(H)** Branch2_layer2 (5 × 5 conv), **(I)** Branch3_layer2(7 × 7 conv).

From Figures 13A–C, we can see that after the head convolution layer with different convolution kernels in three branches, the feature maps of the tanker image have different attention. The feature map from Branch3_Head with a larger receptive field is obviously coarser than that of Branch2_Head and Branch1_Head, which have smaller receptive fields. It shows that the feature map from Branch3_Head is more concerned with coarse-grained feature extraction, whereas Branch1_Head with the smallest receptive field is clearer in detail and is obviously more concerned with the fine-grained feature extraction.

Figures 13D–F show the feature maps of the tanker image after processing by the layer1 of each branch. Branch1_layer1 obviously retains more details and pays more attention to the fine-grained features of the ship. Branch1_layer2 has fewer details than Branch1_layer1 and focuses more on the moderate scale features such as the superstructures of the tanker. Branch1_layer3 focuses on the contours of the ship and the coarse-grained features of the hull structure of ships.

Figures 13G–I shows the feature maps of the tanker image after the layer2 of each branch. It can be clearly seen that there are huge differences between the feature maps after all the convolution layers of each branch. The feature map of *Branch1* is clear and focuses on fine-grained features such as ship equipment. The feature map of *Branch2* focuses on the superstructures of the tanker. The feature map of *Branch3* pays attention to contours and the hull structure of the tanker.

By comparing the feature maps of different convolution of the three branches, we demonstrate the effectiveness of using multi-scale convolutional branch parallelization for ship images.

We also compared the feature maps of each convolutional layer of single branch. Taking Figures 13A,D,G as an example, after the processing of CNN *Head, Layer1*, and *Layer2*, feature maps are gradually abstracted from specific low dimension to high dimension, which is effective for image feature extraction. Besides, from Figures 13D–F, we observe that there is redundant clutter in part of the output from *Layer1* of each branch. Therefore, the reduced numbers of channels in *Layer2* can filter redundant feature maps from the previous layer. This verified the effectiveness of changing numbers of channels in the network.

## Conclusion

We found that the differences between different categories of ships are mainly focused on the hull structures, whereas the differences between different ships of the same category are concentrated in parts such as ship equipment and superstructures. Therefore, we used convolutional kernels with larger receptive fields to extract coarse-grained features such as the hull structure of different categories of ships and used convolutional kernels with smaller receptive fields to extract fine-grained features of different ships of the same category. In this paper, we proposed the SMS-PCNN model for the recognition and classification of ship images. The SMS-PCNN model extracted features of image in different sizes by parallelizing convolutional branches with different receptive fields. In addition, the results of experiments showed that the SMS-PCNN model achieved 84.79% accuracy on a ship dataset containing samples of 12 ship categories. We also conducted a series of comparison experiments to test the performance of algorithms using other state-of-the-art approaches. The results showed that comparing with the Resnet-50, Alexnet, VGG-16, and Resnet-18 models, the SMS-PCNN model improved the accuracy by 14.7–20.97%. We also experimented with the Branch1-CNN model, and the results showed that the SMS-PCNN model with parallel structure improved the accuracy by 9.26% compared with the single-branch model, thus proving that the multi-branch parallel structure outperformed the single-branch for the ship image feature extraction.

The SMS-PCNN model uses three tricks to improve the performance, including enhancing specific ship dataset applications, improving training learning rate, and avoiding overfitting. The experiments showed that the model achieves an accuracy of 77.28% without using any tricks, which is 7.19% higher than Resnet-18. This accuracy is the highest among state-of-the-art approaches. It proved the superiority of the model design principles and the basic framework structure. To verify the effect of optimization tricks on the SMS-PCNN model, we performed a series of ablation experiments. When trick1 was added on the basic SMS-PCNN model, the accuracy increased by 2.16%. When adding trick2 to the SMS-PCNN model with trick1, the accuracy increased by 3.8%. Finally, when adding trick3 to the SMS-PCNN model with trick1 and trick2, the accuracy increased by 1.55%. The ablation experiments validated the effectiveness of the three tricks we used for the ship dataset.

## Data Availability Statement

The original contributions presented in the study are included in the article/supplementary material, further inquiries can be directed to the corresponding author/s.

## Author Contributions

FW designed research, performed research, analyzed data, and wrote the paper. HL supervised the entire research process. YZ was involved in writing part of the manuscript. QX and RZ were involved in collected the data. All authors contributed to the article and approved the submitted version.

## Funding

This study was funded by Naval University of Engineering, College of Electric Engineering.

## Conflict of Interest

The authors declare that the research was conducted in the absence of any commercial or financial relationships that could be construed as a potential conflict of interest.

## Publisher's Note

All claims expressed in this article are solely those of the authors and do not necessarily represent those of their affiliated organizations, or those of the publisher, the editors and the reviewers. Any product that may be evaluated in this article, or claim that may be made by its manufacturer, is not guaranteed or endorsed by the publisher.
